# *In vitro* micronucleus assay: Method for assessment of nanomaterials using cytochalasin B

**DOI:** 10.3389/ftox.2023.1171960

**Published:** 2023-04-26

**Authors:** Christopher S. Farabaugh, Shareen Doak, Shambhu Roy, Rosalie Elespuru

**Affiliations:** ^1^ Charles River Laboratories, Skokie, IL, United States; ^2^ Institute of Life Science 2, Swansea University Medical School, Singleton Park, Swansea, Wales, United Kingdom; ^3^ Inotiv, Rockville, MD, United States; ^4^ Center for Devices and Radiological Health, U.S. Food and Drug Administration, Silver Spring, Columbia, MD, United States

**Keywords:** nanomaterial, genotoxicity, micronucleus, MNvit, hazard id, clastogenicity

## Abstract

The *in vitro* micronucleus (MNvit) assay is used to evaluate the aneugenic and clastogenic potential of a test material based upon its ability to induce micronuclei in the cells. This protocol is provided for testing of nanomaterials (NM) with standard cell lines in the absence of metabolic activation. The use of cytochalasin B (CytoB) and the analysis of binucleated cells in the cytokinesis-block version of the micronucleus assay ensures that cells analyzed have undergone cell division, which is required for expression of DNA damage and micronucleus formation. Issues specific to NM that were problematic with standard test methods are addressed, including test system choice, dose selection, test material exposures, CytoB timing, cytotoxicity determination, and DNA damage expression time. A step-by-step protocol for *in vitro* micronucleus assessment of NM is provided.

## Graphical Abstract

**Figure:**
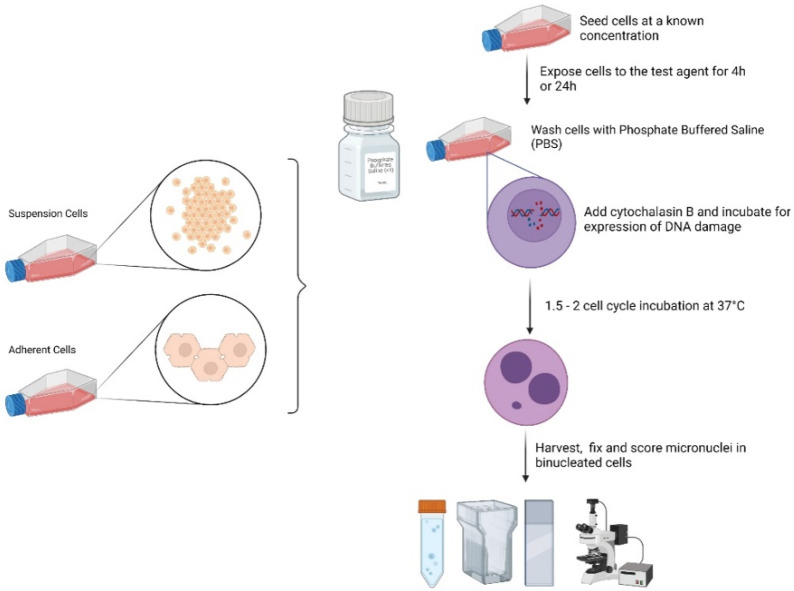
Figure created using BioRender.com by Dr. Michael J. Burgum (*In Vitro Toxicology* group, Swansea University).

## 1 Introduction

The methods found in a set of 4 papers in Frontiers in Toxicology are a follow-up to the analysis and critique of the literature on genotoxicity assessment of nanomaterials (NMs) by an international group working together via the GTTC (Genetic Toxicology Testing Committees) of the Health and Environmental Sciences Institute (HESI) (Elespuru et al., 2018). The *in vitro* Micronucleus Assay is the most common *in vitro* method for genotoxicity assessment of clastogenic, or large-scale DNA damage induced by NMs. Micronuclei are detected as DNA fragments with a nuclear membrane that are not connected to the spindle. The assay recommended here is the cytokinesis-block version of the micronucleus assay. Cytochalasin B (CytoB) prevents the separation of divided cells. Micronuclei are counted in the resulting binucleate cells, ensuring that cells analyzed have undergone DNA replication and expression of DNA damage processing. This obviates the need to experimentally determine optimal and valid timing of micronucleus formation post NM treatment. Interactions with the spindle that could lead to the loss of individual chromosomes (aneugenicity) may be detected as micronuclei in this assay as well. However, specific detection of aneugens (agents causing whole chromosome loss) requires additional methods not described here. It should be noted that the common features of approaches to sample preparation, data analysis and data interpretation are found in the accompanying Common Considerations paper (Elespuru et al., 2022). Additional information is found in OECD 487, OECD, (2016) [2].

## 2 Test system

### 2.1 Cell type

Various immortalized cell lines (TK6, CHO-WBL, CHO-K1, CHL, V79 and L5178Y), are suitable for this test as defined in the OECD Test Guideline 487 (2016) section 14, and in Lorge et al. (2016). Other cell lines such as HepG2 and HepaRG, as well as 3D organoids may be suitable but have not been extensively validated. Cultured cells should be proliferating during the treatment. Whenever possible, cell lines with stable karyotypes should be selected as the test system. Cells should be obtained from a reliable supplier (define the resource and designation) and propagated in the testing laboratory using culture media recommended by the supplier, to make a sufficient batch of cells for future use. Cell lines should be routinely tested for *mycoplasma* using a suitable detection method. Cell culture examination should be undertaken to assess cell health and sterility at regular intervals. Cells should only be used to certain passage number; for example, TK6 cells should be used for no more than 20 to 25 passages, CHO-WBL and CHO-K1 should be used for no more than 20 passages.

### 2.2 Cell Method

#### 2.2.1 Preparation of target cells

i. If adherent cell lines are used (e.g., CHO-WBL, HepG2), the cultures should be incubated in cell culture flasks, e.g., 75 cm^2^, under standard conditions (37°C in a humidified atmosphere of 5% CO_2_ in air) for 16–24 h prior to treatment to establish the monolayer culture. Exponentially growing cells should be verified by population doubling measurements. Cells should be removed from the monolayer culture (e.g., with trypsin) and seeded at 1.0 × 10^5^ cells/mL in fresh complete medium to initiate treatment. Seeding density of cells should be such that at the harvesting of cells, the monolayer confluence will be 70%–80%.
ii. If suspension cell lines are used (e.g., TK6 or L5178Y cells), the exponentially growing cells should be soft pelleted at 200 × g for 10 min; media is aspirated, and the cells are suspended in fresh complete culture medium in tubes or flasks at 1 × 10^5^ cells per mL to initiate the treatment.

#### 2.2.2 Metabolic activation system

Generally, it is not necessary to test NMs in the presence of exogenous metabolizing enzymes (S9). However, if the test material is known or suspected to undergo metabolic transformation, an exogenous metabolic activation system should be used as described in the accompanying Common Considerations paper. Refer to this paper for the recipe for metabolic activation mix [https://doi.org/10.3389/ftox.2022.859122].

## 3 Preparation of NM for testing

See Common Considerations for approaches to NM characterization. NMs and nanoparticles (NPs) should be prepared for testing, e.g., by sonication of particles and suspended in a vehicle compatible with the test system, such as cell culture medium, DMSO, or saline. Cell culture media preparations can be added directly to the cells, whereas saline requires a 1:10 dilution and non-polar solvents such as DMSO require a 1:100 dilution into the cells.

## 4 Preliminary experiments

### 4.1 Dose-range determination

If cytotoxicity information on the test material is not available, a range-finding cytotoxicity assay should be conducted. The range-finding assay is an abbreviated method (not requiring duplicate samples or positive controls) designed to test a wide range of test article doses to select doses for the definitive study. Both short (4h) and long (1.5–2 cell cycles; times vary by cell type) exposures should be considered. The longer exposure may be more relevant for NM as not all NM can be internalized into the target cell during the short (4h) exposure period. The test article should be evaluated starting at the highest concentration that can be prepared and administered as a workable suspension, where possible, absent of agglomeration or aggregation, and extending into a lower dose range, e.g., at half log intervals, with varying levels of cytotoxicity. Acceptable dose levels for the definitive assay are based on cytotoxicity measured at the end of treatment (cytokinesis-blocked proliferation index, CBPI).

If the test material alters the test medium, e.g., changes the pH, osmolality, or precipitation profile in culture, treatment doses affecting those parameters should be determined in the range-finding study. Those doses can be avoided or included, and the information can be used in the interpretation of results. If a dose range-finding study is not performed, those parameters can be assessed in the definitive experiment.

#### 4.1.1 Dose: test materials exposure

For the dose-response, the test article should be serially diluted into aliquots of a freshly prepared proliferating cell culture (e.g., 5 mL in vented 25 cm^2^ flasks). The volume of test sample added to the cells should be the same at each dose level, e.g., a 1:10 dilution. If feasible, dilutions should be made in culture medium. The cultures are then incubated for 1-1.5 cell divisions at 37°C with 5% CO_2_ in humidified air.

#### 4.1.2 Washing

At the end of the treatment time, the cells are harvested by centrifugation at 200 *×* g for 10 min. Cells are washed with PBS 3 times to remove the NM by centrifuging at 200 *×* g 3 times for 10 min each time, and then fresh culture medium is added. An adherent cell type may be preferred if the test material precipitates at several concentrations and is difficult to remove.

#### 4.1.3 Addition of cytochalasin B

Cytochalasin B (CytoB) is added to each culture after completing the washing procedure. Final concentrations of CytoB vary according to cell type (3–6 μg/mL). The cells are returned to the incubator for the duration of the expression time, 1-2 cell cycle times. If the test material is suspected or known to inhibit cell division, the recovery period could be extended up to 3 to 4 times that required for one cell cycle in the absence of NM. The recovery time also may vary with the chemical properties of the test material.

#### 4.1.4 Harvest

The cultures are removed from the incubator after the expression period and visually observed for changes to color of the media, cell lysis and/or the presence of a NM precipitate or agglomerate that was not present at the beginning of the treatment. Cells are evaluated for cytotoxicity to choose a dose-range for the definitive experiment.

#### 4.1.5 Cytotoxicity evaluation (see also OECD 487)

At least 1,000 cells (500 cells per culture, in case of replicate cultures), if possible, should be evaluated to determine the cytochalasin B proliferation index (CBPI) at each dose level and the control. The CBPI is determined using the following formula (from OECD TG487, Annex 2):

$$CBPI=\left(\frac{\left(1x\;Mononucleated\;cells\right)+\left(2\;x\;Binucleated\;cells\right)+\left(3\;x\;Multinucleated\;cells\right)}{Total\;number\;of\;cells\;scored}\right)$$

$$\%\;Cytostasis\;\left(cytotoxicity\right)=100-100\left\{\left(CBPI_{T}-1\right)÷\left(CBPI_{C}-1\right)\right\}$$

T = test article treatment culture.C = vehicle control culture.

a. For adherent cells: Trypsin is generally used to detach adherent cells from the flask. Prior to trypsinization, flasks should be carefully observed under a phase contrast microscope for rounded cells. Dividing cells in the mitotic phase are generally round and loosely attached to the flask surface. These cells can easily detach and be lost during the washing procedure. Thus, the washing medium should be collected, centrifuged and the pellet added back to the culture.
b. For suspension cells: When using suspension cultures, extra attention should be paid to the effectiveness of the washing procedure in removing NM particles from the cultures, if visible. Care in choosing doses also minimizes problems related to residual NM during the gene expression phase.

## 5 Micronucleus assay

The definitive micronucleus assay is conducted as described for the preliminary range-finding experiments with at least four concentrations of the test article, and concurrent solvent and positive controls in duplicate cultures.

### 5.1 Test material exposure (note the results in the preliminary experiments, section 4.1 and 4.1.1)

At least one of the following criteria should be met in order to select the highest concentration for micronucleus scoring:

1. 10 mM, 2 mg/mL or 2 µL/mL, whichever is the lowest (OECD 487) (may not be practical for NM due to likelihood of agglomeration and/or sedimentation at high dose levels)
2. Produces target cytotoxicity when possible (55% ± 5%)
3. Highest dose without aggregation (may be a more relevant indicator for NM)

The lower concentrations should include, when possible, one that has moderate toxicity, e.g., 25%, and one that is relatively non-toxic, e.g., 10%.

#### 5.1.1 Positive controls

Concurrent chemical positive controls should be used in each treatment condition. Currently, there are no agreed upon NM positive controls, but the chemicals below can be used to demonstrate appropriate performance of the assay.

Clastogenic positive controls without metabolic activation:

Methyl methanesulphonate (CAS No.: 66-27-3), 10 µg/mL

Mitomycin C (CAS No.: 50-07-7), 0.2 µg/mL

4-Nitroquinoline-N-Oxide (CAS No: 56-57-5), 0.5 µg/mL

Cytosine arabinoside (CAS No: 147-94-4), 0.5 µg/mL

### 5.2 Washing

(Follow the method in Section 4.1.2).

### 5.3 Addition of cytoB

(Follow the method in Section 4.1.3).

### 5.4 Harvest and fixation of cells for micronucleus determination

Cells are examined for any change in color of the medium, cell lysis, or precipitate. Aliquots of cells from each sample are removed and combined with an equal volume of hypotonic KCl (0.075M) and gently mixed by inversion. An aliquot of one-tenth volume of cold fixative (methanol/glacial acetic acid: 25:1) is added and gently mixed. Cells are harvested by centrifugation (200 × g for 10 min) and the supernatant is aspirated. An aliquot of additional fixative is added equal to the original cell volume removed from the culture. Slides may be made from the cell suspensions and the residue of the fixed cultures may be stored at 4°C.

## 6 Slide preparation

### 6.1 Dosing selection for slide analysis

At least three test article concentrations plus concurrent negative and positive controls should be selected for evaluation. The highest concentration should be selected as described in section 5.1.

### 6.2 Coding

Slides should be coded and scored for the presence of micronuclei by a person blinded to sample identity.

### 6.3 Dropping slides

After fixation, cells are mixed briefly and drops are applied gently to slides with a Pasteur pipette and air dried.

### 6.4 Staining

The slides are stained for a time appropriate to attain suitable staining in the cells with Giemsa, DifQuik, Acridine Orange, or other nuclear stain.

### 6.5 Coverslipping

Coverslips may be mounted onto slides using a permanent mounting medium once slides have dried, or a wet mount may be used.

## 7 Evaluation of micronuclei

Based on the cytotoxicity profile, at least three concentrations with test article treatment should be evaluated for micronucleus induction. Slides should be coded and scored for the presence of micronuclei by a person blinded to sample identity. Whenever possible, a minimum of 2000 binucleated cells (if possible, 1,000 cells from each replicate culture) should be evaluated. Slides may be evaluated manually or with an automated device like an image analyzer that has been optimized for evaluation of the cell type under investigation. The protocol for slide fixation and analysis may be different for image analysis.

Micronuclei in a binucleated cell will be recorded if they meet the following criteria (Fenech, 2007):

1. They have the same staining characteristics as the main nucleus
2. They are separate from the main nuclei or just touching (no cytoplasmic bridges)
3. They have a regular shape and approximately 1/3 or less than the diameter of the main nucleus

The induction of micronuclei should be presented as % micronucleated cells irrespective of the number of micronuclei in a cell. For example, cells with two or more micronuclei will be considered as one aberrant cell.

To help judge dose-dependence of the effect, the Cochran–Armitage trend test (*p* < 0.05) can be used in the overall judgment of the effect. Other statistical analysis such as one-way ANOVA with Dunnet’s can be used.

## 8 Evaluation of uptake

See also the Common Considerations paper.

Uptake of the NMs into the cells of the test system should be documented and the location of particles determined within the cells if feasible (i.e., nucleus or cytoplasm), particularly in the demonstration of a negative effect. Transmission electron microscopy remains the gold standard for determining the sub-cellular localization of nanomaterials upon internalization into cells; it should however be noted that this is a qualitative method for uptake analysis (Hondow et al., 2011; Singh et al., 2012). In some cases, results may be positive in the absence of uptake, which could reflect breakdown of the material in the test environment and/or the release of diffusible genotoxins.

## Author disclaimer

This article has been reviewed by the agencies and organizations of the authors and approved for publication. The views expressed in the manuscript do not necessarily reflect the policy of these agencies and organizations. The findings and conclusions in this article have not been formally disseminated by the Food and Drug Administration and should not be construed to represent any agency determination or policy. The mention of commercial products, their sources, or their use in connection with material reported herein is not to be construed as either an actual or implied endorsement of such products by the Department of Health and Human Services.

## References

[B1] Elespuru R. K. Doak S. H. Collins A. Dusinska M. Pfuhler S. Manjanatha M. (2022). Common Considerations for genotoxicity assessment of nanomaterials. Front. Toxicol. 4, 859122. 10.3389/ftox.2022.859122 35686044 PMC9171035

[B2] Elespuru R. Pfuhler S. Aardema M. J. Chen T. Doak S. H. Doherty A. (2018). Genotoxicity assessment of nanomaterials: Recommendations on best practices, assays, and methods. Toxicol. Sci. 164 (2), 391–416. 10.1093/toxsci/kfy100 29701824

[B3] Fenech M. (2007). Cytokinesis-block micronucleus cytome assay. Nat. Protoc. 2 (5), 1084–1104. 10.1038/nprot.2007.77 17546000

[B4] Hondow N. Harrington J. Brydson R. Doak S. H. Singh N. Manshian B. (2011). STEM mode in the SEM: A practical tool for nanotoxicology. Nanotoxicology 5 (2), 215–227. 10.3109/17435390.2010.535622 21090920

[B5] Lorge E. Moore M. M. Clements J. O'Donovan M. Fellows M. D. Honma M. (2016). Standardized cell sources and recommendations for good cell culture practices in genotoxicity testing. Mutat. Res. Genet. Toxicol. Environ. Mutagen 809, 1–15. 10.1016/j.mrgentox.2016.08.001 27692294

[B6] OECD (2016). “Test No. 487,” *in Vitro* mammalian cell micronucleus test (Paris, France: OECD).

[B7] Singh N. Jenkins G. J. Nelson B. C. Marquis B. J. Maffeis T. G. Brown A. P. (2012). The role of iron redox state in the genotoxicity of ultrafine superparamagnetic iron oxide nanoparticles. Biomaterials 33 (1), 163–170. 10.1016/j.biomaterials.2011.09.087 22027595

